# G-Protein Inwardly Rectifying Potassium Channel 1 (GIRK1) Knockdown Decreases Beta-Adrenergic, MAP Kinase and Akt Signaling in the MDA-MB-453 Breast Cancer Cell Line

**DOI:** 10.4137/bcbcr.s629

**Published:** 2008-03-26

**Authors:** Michael W. Hance, Madhu S. Dhar, Howard K. Plummer

**Affiliations:** 1 Molecular Cancer Analysis Laboratory, Department of Pathobiology, College of Veterinary Medicine, University of Tennessee, Knoxville, TN 37996-4542, U.S.A; 2 Molecular Cancer Analysis Laboratory, Department of Pathobiology, and Department of Large Animal Clinical Sciences, College of Veterinary Medicine, University of Tennessee, Knoxville, TN 37996-4542, U.S.A

**Keywords:** GIRK, siRNA, breast cancer, real-time PCR, MAP kinase, Akt, beta-adrenergic

## Abstract

Previous data from our laboratory have indicated that there is a functional link between the beta-adrenergic receptor signaling pathway and the G-protein inwardly rectifying potassium channel (GIRK1) in breast cancer cell lines and that these pathways are involved in growth regulation of these cells. To determine functionality, MDA-MB-453 breast cancer cells were stimulated with ethanol, known to open GIRK channels. Decreased GIRK1 protein levels were seen after treatment with 0.12% ethanol. In addition, serum-free media completely inhibited GIRK1 protein expression. This data indicates that there are functional GIRK channels in breast cancer cells and that these channels are involved in cellular signaling. In the present research, to further define the signaling pathways involved, we performed RNA interference (siRNA) studies. Three stealth siRNA constructs were made starting at bases 1104, 1315, and 1490 of the GIRK1 sequence. These constructs were transfected into MDA-MB-453 cells, and both RNA and protein were isolated. GIRK1, β_2_-adrenergic and 18S control levels were determined using real-time PCR 24 hours after transfection. All three constructs decreased GIRK1 mRNA levels. However, β_2_ mRNA levels were unchanged by the GIRK1 knockdown. GIRK1 protein levels were also reduced by the knockdown, and this knockdown led to decreases in beta-adrenergic, MAP kinase and Akt signaling.

## Introduction

Breast cancer is the most frequently diagnosed cancer in women and the second leading cause of cancer death for women (American Cancer Society, 2007). There were an estimated 178,480 new cases of invasive breast cancer in 2007 in the U.S.A., making breast cancer responsible for 26% of new cancers (American Cancer Society, 2007). Worldwide, an estimated 1 million new cases occur each year, with a higher incidence in Western countries (Baselga and Norton, 2002). Established risk factors for breast cancer include age, increased hormone exposure, estrogen, alcohol consumption, and family history, as well as many other factors (Baselga and Norton, 2002). Approximately 40% of primary human breast cancer tissues have shown expression of mRNA that encodes a G-protein-coupled inwardly rectifying potassium channel (GIRK1), and this expression of GIRK1 was associated with a more aggressive clinical behavior (Stringer et al. 2001). Previous data from our laboratory has indicated that a functional link exists between the GIRK1 channel and the beta-adrenergic receptor pathway in breast cancer cell lines, and these pathways were involved in growth regulation of these cells (Cakir et al. 2002; Plummer et al. 2004). The estrogen receptor positive (ER (+)) cell lines MCF-7, MDA-MB-361, and ZR-75-1 and the ER negative (−) cell line MDA-MB-453 expressed mRNA for the GIRK1 channel while the ER (−) cell lines MDA-MB-468 and MDA-MB-435S did not express GIRK1 (Plummer et al. 2004). Gene expression data indicated that mRNA for GIRK2 was observed in all cell lines except ZR-75-1 and MDA-MB-435S and indicated that mRNA for GIRK4 was also observed in all cell lines (Plummer et al. 2004).

GIRK protein expression was also identified in breast cancer cell lines. Expression of GIRK1 at the indicated molecular weight (MW) (62 kDa) was seen in cell lines MDA-MB-453 and ZR-75-1 (Dhar and Plummer, 2006). In addition, GIRK1 expression was seen at a lower MW (40–42 kDa) in MDA-MB-361, MDA-MB-468, MCF-7, ZR-75-1, and MDA-MB-453 cell lines. GIRK2 expression was seen in MDA-MB-453, MDA-MB-468, MCF-7, and ZR-75-1, while GIRK4 protein expression was seen in all six cell lines tested. Both GIRK2 and GIRK4 were at the indicated MW (Dhar and Plummer, 2006). This was the first report indicating GIRK protein expression in breast cancer cells and the first report of GIRK protein expression outside its normal tissues of origin (heart and brain). Ethanol has been found to open G-protein inwardly rectifying potassium channels (GIRKs) in both heart and brain cells (Kobayashi et al. 1999). In addition, treatment of MCF-7 breast cancer cells with ethanol increased ERK1/2 activities and resulted in subsequent increased cell growth (Izevbigie et al. 2002). To determine functionality of GIRK channels in breast cancer cells, MDA-MB-453 cells were stimulated with ethanol in our research. Decreased GIRK1 protein levels were seen after treatment with 0.12% ethanol in MDA-MB-453 breast cancer cells (Dhar and Plummer, 2006). Transfection of GIRK1 or GIRK4 plasmids increased GIRK1 protein expression and decreased gene expression in MDA-MB-453 breast cancer cells (Dhar and Plummer, 2006). This previous data indicates that functional GIRK channels exist in breast cancer cells and that they are involved in cellular signaling (Dhar and Plummer, 2006).

Serum-free media also decreased GIRK protein expression, possibly due to lack of estrogen in the media (Dhar and Plummer, 2006). Previous research has indicated that estrogen may have effects on potassium channels. Specifically, activation of potassium channels may be involved in the effects of 17-β-estradiol in rat mesenteric artery rings (Tsang et al. 2003). In addition, 17-β-estradiol can modulate GIRK channel activation in the brain, and this modulation is blocked by protein kinase A (PKA) and protein kinase C (PKC) inhibitors (Kelly et al. 2003). Estrogen induced a rapid and irreversible augmentation of potassium currents in MCF-7 cells (Coiret et al. 2005). Two potassium channel blockers (amiodarone and dequalinium) also potentiated the growth inhibitory effects of the breast cancer drug tamoxifen in two breast cancer cell lines (Abdul et al. 2003). If estrogen affects potassium channels, then blockage of potassium channels would be a different and innovative method for cancer treatment, especially in ER (−) tumors.

To further define the signaling pathways involved, we performed RNA interference (siRNA) studies on ER (−) MDA-MB-453 cells, which we have used extensively in previous research in our laboratory. RNA interference is a more effective and efficient technique for gene knockdown than other techniques (Cullen, 2006). This technique could also possibly be used for drug development in cancer (Mousses, 2003). Our previous research has used the MDA-MB-453 cell line (Cakir et al. 2002; Plummer et al. 2004; Dhar and Plummer, 2006), so these siRNA experiments were performed in this cell line for consistency. Multiple investigators have used one breast cancer cell line for RNA interference studies (Zivadinovic et al. 2005; Santra et al. 2000; Lu and Serrero, 2001). In this study, three stealth siRNA constructs were transfected into the MDA-MB-453 breast cancer cell line, and protein was isolated. GIRK1 knockdown led to decreases in β-adrenergic, MAP kinase and Akt signaling.

## Materials and Methods

### Cell culture

The human estrogen non-responsive MDA-MB-453 breast cancer cell line was purchased from the American Type Culture Collection (Rockville, MD, U.S.A.) and was maintained in RPMI 1640 medium supplemented with fetal bovine serum (10%, v/v), L-glutamine (2 mM), 100 U/ml penicillin, and 100 μg/ml streptomycin at 37 °C in an environment of 5% CO_2_.

### Real-time PCR

RNA isolation and real-time PCR were done as previously described (Plummer et al. 2004, 2005). Briefly, real-time PCR was done using the Cepheid smart cycler. The GIRK1 primers for real-time PCR are forward 5′-ctctcggacctcttcaccac-3′ and reverse 5′-gccacggtgtaggtgagaat-3′ (bases 398–477, Genbank Accession # NM_002239), and the internal TaqMan probe is 6-FAM-tcaagtggcgctggaacctc-TAMRA (bases 429–449). The real-time PCR conditions for GIRK1 were 95 °C for 120 seconds, followed by 45 cycles of 95 °C, 15 seconds; 62 °C, 10 seconds; and 72 °C, 15 seconds. 18S rRNA detection reagents (Eurogentec, San Diego, CA, U.S.A.) were used for normalization of the data.

### siRNA

Three stealth siRNA constructs (Invitrogen, Carlsbad, CA, U.S.A.) were made starting at bases 1104, 1315, and 1490 of the GIRK1 sequence (GenBank # NM_002239). These constructs are: 1104 sense─gga aac aac ugg gau gac uug uca a; 1104 antisense─uug aca agu cau ccc agu ugu uuc c (GC content 44%); 1315 sense─cca gcc aua acu aac agc aaa gaa a; 1315 antisense─uuu cuu ugc ugu uag uua ugg cug g (GC content 40%); 1490 sense─acu ugc cca uga aac uuc aac gaa u; 1490 antisense─auu cgu uga agu uuc aug ggc aag u (GC content 40%). In addition, the low GC RNAi negative control (35%–45% GC) was used as recommended by the manufacturer (Invitrogen). These constructs (200 pMol) were transfected into the MDA-MB-453 breast cancer cell lines using lipofectamine 2000 (Invitrogen).

### Western blots

Cells were harvested, and membrane and total proteins were isolated as previously described (Plummer et al. 2004, 2005; Dhar and Plummer, 2006). Antibodies for GIRK1 (Lifespan Biosciences, Seattle, WA, U.S.A.), β_2_ (Santa Cruz, Santa Cruz, CA, U.S.A.), CREB, phosphoCREB (Upstate, Lake Placid, NY, U.S.A.), Akt, phosphoAkt, ERK, and phosphoERK (Cell Signaling, Danvers, MA, U.S.A.) were used in these studies. Immunoblot analysis was carried out using standard procedures as described previously (Plummer et al. 2004, 2005; Dhar and Plummer, 2006). Briefly, 20 μg of proteins were resolved on 12% SDS-PAGE, transferred to nitrocellulose membranes, blocked for 1 hour, and immunoblotted overnight with the specific primary antibodies. Primary antibodies were detected with horseradish peroxidase-coupled secondary antibody and enhanced chemiluminescence (Pierce, Rockford, IL, U.S.A.). In all Western blots, membranes were additionally probed with an antibody for GAPDH (Santa Cruz) to ensure equal loading of protein between samples. GAPDH has been previously used as a loading control (Hambsch et al. 2005). Actin was not used because GIRKs may affect actin (Huang et al. 1998; Nikolov and Ivanova-Nikolova, 2004). Protein bands on the immunoblots were quantitated using Scion Image software, release Alpha 4.0.3.2 (http://www.scioncorp.com) using three separate measurements of each band on the gel.

## Results

**Figure 1.**
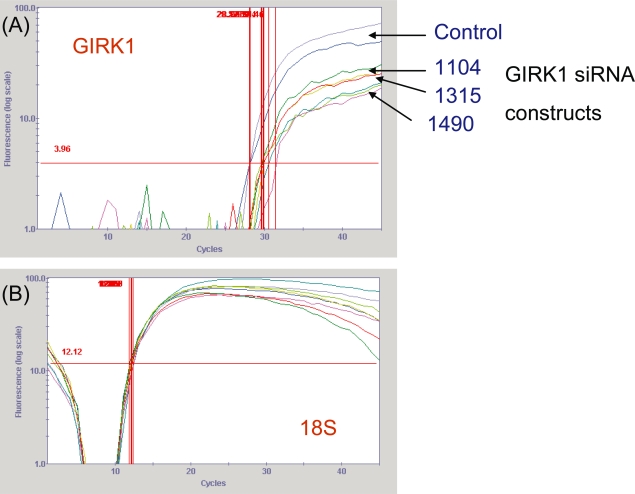
**Real-time PCR of MDA-MB-453 cells transfected with GIRK1 stealth siRNA.** MDA-MB-453 breast cancer cells were transfected with 3 stealth siRNA constructs starting at bases 1104, 1315, and 1490 of the GIRK1 sequence (GenBank # NM_002239) (Invitrogen). RNA was isolated 24 hours after transfection and cDNA was made. (**A**) Real-time PCR graph of MDA-MB-453 cells transfected with three GIRK1 siRNA constructs (1104, 1315, 1490). All three siRNA constructs produced noticeable GIRK1 gene knockdown. Numbers to the right of graph indicate the three separate constructs. (**B**) Real-time PCR graph of 18S genomic control levels of MDA-MB-453 cells transfected with three GIRK1 siRNA constructs. No changes were noted in 18S levels indicating that the changes seen in graph (**A**) are due to the experimental effects. These are graphs representative of two separate experiments. None of the constructs altered β-adrenergic gene expression (data not shown).

Three stealth siRNA constructs (Invitrogen) were made starting at bases 1104, 1315, and 1490 of the GIRK1 sequence (GenBank # NM_002239) to knockdown GIRK1 levels in the MDA-MB-453 breast cancer cell line used extensively in previous research in our laboratory (Plummer et al. 2004; Dhar and Plummer, 2006). These constructs were transfected into MDA-MB-453, and RNA was isolated 24 hours after transfection. GIRK1, β_2_-adrenergic and 18S control levels were determined using real-time PCR. All three constructs noticeably decreased GIRK1 mRNA levels (Fig. 1). Data are presented as GIRK1 C_T_ value minus 18S C_T_ value (n = 2): control: 15.9; 1104: 17.93 (88.6% of corrected control C_T_); 1315: 17.60 (90.3% of corrected control C_T_); and 1490: 18.63 (85.3% of corrected control C_T_). However, β_2_ mRNA levels were unchanged by the GIRK1 knockdown (data not shown).

**Figure 2.**
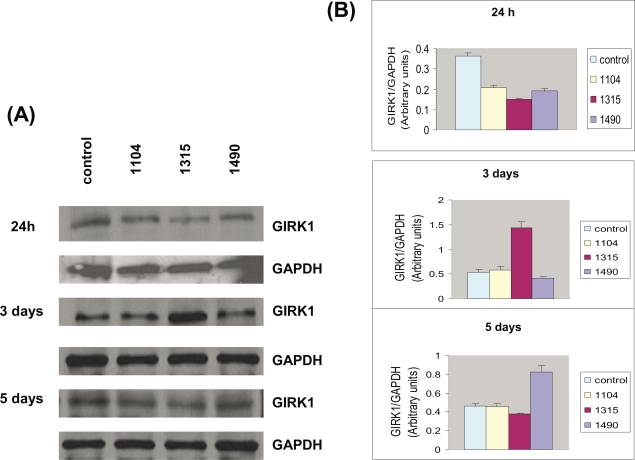
**GIRK1 protein expression of MDA-MB-453 cells transfected with GIRK1 stealth siRNA.** MDA-MB-453 breast cancer cells were transfected with 3 stealth siRNA constructs (Invitrogen). Enriched membrane protein was isolated 1, 3, and 5 days after transfection by the ReadyPrep protein extraction kit (signal) (Bio-Rad, Richmond, CA, U.S.A). Western blotting was performed, and the membranes were probed with antibodies to GIRK1 and GAPDH. (**A**) Western blots indicating GIRK1 and GAPDH expression of MDA-MB-453 cells transfected with three GIRK1 siRNA constructs (1104, 1315, 1490). Samples were collected at 24 hours, 3 days and 5 days. (**B**) Graphs indicate densitometry of the Western blots. The densitometry of GIRK1 was divided by the densitometry of the control GAPDH (N = 3). All three siRNA constructs reduced GIRK1 protein levels 24 hours after transfection. These reductions were reversed by 3–5 days, and in the case of one construct, increased over control levels. The bands are consistent with the expected size: GIRK1 (40–42 kDa; Dhar and Plummer 2006); GAPDH (37 kDa). These are gels representative of two separate experiments.

These three siRNA constructs were also transfected into additional MDA-MB-453 cells, and protein was isolated after 24 hours, 3 days and 5 days after transfection. Enriched membrane protein was isolated, Western blotting was performed, and the membranes were probed with antibodies to GIRK1. All three siRNA constructs reduced GIRK1 protein levels 24 hours after transfection (Fig. 2). At 24 hours, the GIRK1 protein levels were 57.1% of control levels for the 1104 construct, 41.2% of control levels for the 1315 construct and 52.1% of control levels for the 1490 construct. These reductions were reversed by 3–5 days, and in the case of one construct, increased over control levels (Fig. 2). At 3 days, the GIRK1 protein levels were 109.7% of control levels for the 1104 construct, 267.8% of control levels for the 1315 construct and 78.1% of control levels for the 1490 construct. At 5 days, the GIRK1 protein levels were 99.0% of control levels for the 1104 construct, 81.1% of control levels for the 1315 construct and 178.8% of control levels for the 1490 construct. When the low GC RNAi negative control (Invitrogen) for the GIRK1 stealth siRNA was used, there were no effects on GIRK1 protein expression at either one, three or five days (data not shown).

**Figure 3.**
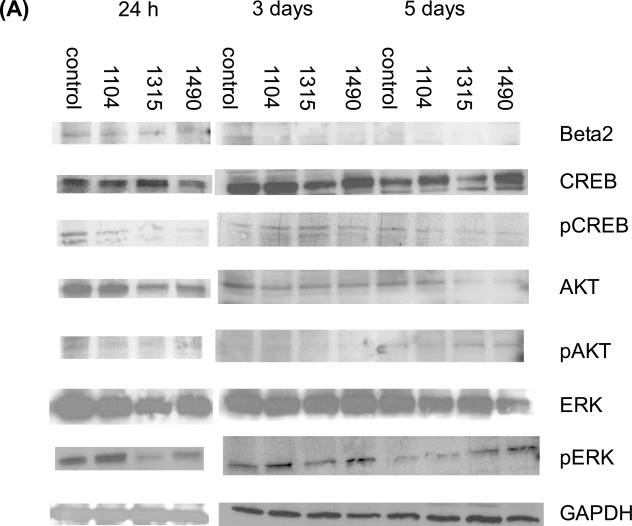

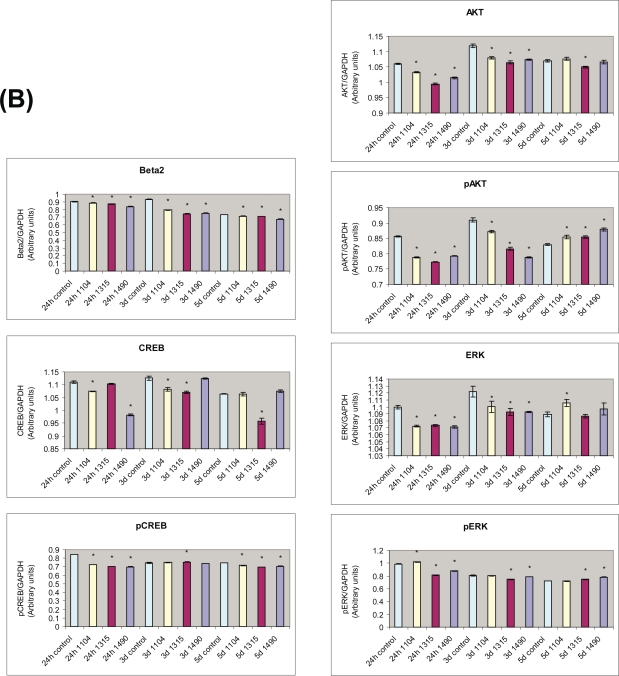
**Protein expression of components of cell signaling pathways in MDA-MB-453 cells transfected with GIRK1 stealth siRNA.** MDA-MB-453 breast cancer cells were transfected with 3 stealth siRNA constructs (Invitrogen). Total protein was isolated 1, 3, and 5 days after transfection by RIPA. Western blotting was performed, and the membranes were probed with antibodies to the intermediates in the signaling pathways. (**A**) Western blots indicating β_2_-adrenergic, CREB, phosphoCREB, Akt, PhosphoAkt, ERK, phosphoERK and GAPDH expression of MDA-MB-453 cells transfected with three GIRK1 siRNA constructs (1104, 1315, 1490). Samples were collected at 24 hours, 3 days and 5 days. (**B**) Graphs indicate densitometry of the Western blots. The densitometry of signaling proteins being measured were standardized by dividing the densitometry of the control GAPDH (N = 3). Data were analyzed by ANOVA followed by the Tukey test. *indicates p < 0.05 from control. β_2_-adrenergic protein expression was reduced from control levels at all three time periods. β_2_-adrenergic phosphorylation was increased at 24 hours, but reduced at 3 days and 5 days by all three constructs (data not shown). CREB protein expression was reduced by two siRNA constructs at 24 hour (1104,1490) and 3 day (1104,1315), but only one construct at 5 days (1315). CREB phosphorylation was reduced by all constructs at 24 hours and 5 days, but increased by one construct at 3 days (1315). Akt protein expression was reduced by all constructs at 24 hours and 3 days, but only one construct at 5 days (1315). Akt phosphorylation was reduced at 24 hours and 3 days, but increased at 5 days for all constructs. ERK protein expression was reduced at 24 hours and 3 days by all constructs, but increased by one construct at 5 days (1104). ERK phosphorylation was increased at 24 hours (1104), but reduced by the other two constructs; reduced by two constructs at 3 days (1315, 1490); and increased by two constructs at 5 days (1315,1490). The bands are consistent with the expected size: β_2_ (68 kDa); CREB/pCREB (43 kDa); Akt/pAkt (60 kDa); ERK/pERK (42–44 kDa); GAPDH (37 kDa). These are gels representative of two separate experiments.

Total protein was also isolated from the GIRK1 stealth siRNA transfections. Western blots underwent electrophoresis with these proteins, and were probed for possible cell signaling pathways that may be affected by GIRK. Our laboratory has previously indicated a correlation between GIRK expression and β-adrenergic signaling (Cakir et al. 2002; Plummer et al. 2004). Activation of the beta-adrenergic signaling pathway may lead to phosphorylation of the cAMP-response element binding protein (CREB) (Daniel et al. 1999). We also wanted to look at other cellular pathways that we hypothesize could be involved in GIRK signaling, such as the MAP kinase pathway or the Akt pathway. In examining these pathways, we found that β_2_-adrenergic protein expression was reduced from control levels from 1–5 days (Fig. 3). β_2_-adrenergic phosphorylation was increased at 1 day, but reduced at 3 days and 5 days by all three constructs (data not shown). CREB protein expression was reduced by two siRNA constructs at 1 day and 3 days, but only one construct at 5 days (Fig. 3). CREB phosphorylation was reduced by all constructs at 1 day and 5 days, but increased by one construct at 3 days (Fig. 3). This data confirms our previous data that the β-adrenergic signaling pathway is correlated with GIRK expression (Cakir et al. 2002; Plummer et al. 2004). ERK protein expression was reduced at 1 day and 3 days by all constructs, but increased by one construct at 5 days (Fig. 3). ERK phosphorylation was increased by one construct (1104) at 1 day, but reduced by the other two constructs; reduced by the same two constructs at 3 days; and increased by the same two constructs at 5 days (Fig. 3). Akt protein expression was reduced by all constructs at 1 day and 3 days, but only one construct at 5 days. Akt phosphorylation was reduced at 1 day and 3 days, but increased at 5 days for all constructs (Fig. 3). These data indicate that both MAP kinase signaling and Akt signaling may be involved in responses to GIRK.

## Discussion

In the present study, GIRK1 mRNA and protein expression both were reduced by use of stealth siRNA for GIRK1. This is the first time GIRK1 knockdown by use of RNA interference has been reported in the literature. This data supports our previous results that there are functional GIRK channels in breast cancer cell lines (Cakir et al. 2002; Plummer et al. 2004; Dhar and Plummer, 2006). Our previous research has repeatedly used the MDA-MB-453 ER (−) cell line (Cakir et al. 2002; Plummer et al. 2004; Dhar and Plummer, 2006), so these siRNA experiments were performed in this cell line for consistency. ER (−) breast cancers have a poorer prognosis than ER (+) cancers (Nagai et al. 1994; Lemieux et al. 1996). Overexpression of GIRK1 mRNA has been identified in tissue samples from approximately 30% of primary human breast cancers tested (Stringer et al. 2001), and this over-expression of GIRK1 was associated with a more aggressive clinical behavior. Therefore, we believe that the MDA-MB-453 cell line is appropriate for these siRNA studies, because their use aids to further elucidate signal differences in ER (−) cancers.

Previous data from our laboratory established a functional link between β-adrenergic signaling and GIRK channels in breast cancer (Cakir et al. 2002; Plummer et al. 2004). Increases in GIRK currents by β-adrenergic stimulation have been reported in adult rat cardiomyocytes and in *Xenopus laevis* oocytes coexpressing β_2_-adrenergic receptors and GIRK1/GIRK4 subunits (Mullner et al. 2000). In addition, in rat atrial myocytes transiently transfected with β_1_ or β_2_ adrenergic receptors, the β-adrenergic agonist isoproterenol stimulated GIRK currents, whereas this stimulation was not seen in non-transfected cells (Wellner-Kienitz et al. 2001). Activation of the β-adrenergic signaling pathway (a prototypic G-protein-coupled receptor (GPCR); Whalen et al. 2007) can lead to phosphorylation of CREB (Daniel et al. 1999). In the present studies, reductions in GIRK1 mRNA and protein expression lead to reductions in the β-adrenergic signaling pathway as evidenced by decreases in β_2_-adrenergic levels and CREB protein levels, confirming and expanding other data from our laboratory (Cakir et al. 2002; Plummer et al. 2004; Dhar and Plummer, 2006). The present studies also indicated that there were no effects of GIRK1 siRNA knockdown on β_2_-adrenergic mRNA expression. It is our hypothesis that the beta-adrenergic system is potentially reduced through non-genomic pathways. Previous investigators have shown that 1 alpha, 25-dihydroxy-vitamin D3 effects on cardiac muscle calcium influx involves non-genomic modulation of the beta-adrenergic signaling pathway (Santillan et al. 1999). In addition, there are non-genomic actions of 17 beta-estradiol on opening Ca^2+^- and voltage-activated potassium channels in lacrimal acinar cells (Suzuki et al. 2004). Maxi-potassium channels are also activated through a non-genomic pathway in MCF-7 breast cancer cells (Coiret et al. 2005). Further research is needed in order to determine the non-genomic mechanism of beta-adrenergic reduction by GIRK1.

We wanted to investigate whether this reduction of GIRK protein levels, possibly mediated through the β_2_-adrenergic GPCR pathway, has effects on other cellular signaling pathways that have been seen in cancer progression. A recent review indicated that many of the transforming events in breast cancer could be mediated by Akt signaling (Liu et al. 2007). In addition, the tobacco carcinogen 4-(methylnitrosamino)-1-(3-pyridyl)-1-butanone (NNK) has been shown to stimulate cell proliferation mediated through Akt signaling (Tsurutani et al. 2005). Our previous work has also indicated that NNK activates the β-adrenergic GPCR signaling pathway in this MDA-MB-453 cell line (Hance et al. 2006). In the present studies, both ERK and Akt protein levels and protein phosphorylation were reduced by GIRK1 knockdown. Akt phosphorylation was reduced at early time periods but increased at 5 days for all constructs (Fig. 3). In these studies, the data indicates that there is gene knockdown, followed by increases in protein expression. It also appears that there are differences in the activities of the three different constructs, and these differences appear to a greater degree 5 days after introduction of the siRNA constructs. It is apparent that in some cases these constructs either are no longer functioning at 5 days, or that there is an over-compensation for some of the constructs. A recent paper has indicated that some of the differences in efficacy of siRNA constructs may be due to accessibility to target sequences (Liao et al. 2008). It could be that after 5 days, the GIRK target has changed. Further research is needed in order to confirm these hypotheses. In other studies, GIRK channel inhibitors inhibited the platelet P2Y(12)-mediated increase in Akt phosphorylation (Shankar et al. 2004). Akt has been shown to be an important mediator in other potassium channels as well. Akt phosphorylation has been shown to be important in actions of ATP-sensitive potassium channels in rats (Goni-Allo et al. 2007). In the present studies, we show a definitive correlation between GIRK function and Akt signaling in the MDA-MB-453 cell line, indicating that GIRK function could be correlated with a cellular signaling pathway that leads to cellular transformation. Blockage of this pathway could then possibly have important therapeutic effects in ER (−) breast cancer. Other investigators have found that in MCF-7 breast cancer cells, insulin-like growth factor-1 increases both activity and expression of human ether-a-go-go potassium channels by stimulation of Akt (Borowiec et al. 2007). These ether-a-go-go potassium channels were also found to be important in mediating cell proliferation in the MCF-7 cells (Borowiec et al. 2007).

MAP kinase has been shown to be a critical mediator of breast cancer cell migration (Ostrander et al. 2007). In cerebellar granule neurons, high potassium levels resulted in sustained activation of MAP kinase, and additionally, insulin-like growth factor activated Akt (Zhong et al. 2004). These same high potassium levels also led to increases in CREB phosphorylation (Zhong et al. 2004). Insulin-like growth factor has been shown to stimulate breast cancer cell migration (Zhang et al. 2005). In addition, blockage of the MAP kinase pathway in turn blocked the effects of PGE(2) on ROMK-like small conductance potassium channels in the distal tubule segment cortical collecting duct cells (Jin et al. 2007). Similar inhibition of Ca^2+^-activated big conductance potassium channels was seen in distal tubule segment principal cells (Li et al. 2006). In the present studies, we show that responses to GIRK signaling in breast cancer are associated with MAP kinase signaling, indicating that GIRK function may be associated with a signaling pathway correlated with cell migration. This observation may offer a significant therapeutic target, since expression of GIRK1 was associated with a more aggressive clinical behavior in human breast cancer tissues (Stringer et al. 2001). In addition, other investigators indicated that GIRK1 mRNA expression was associated with lymph node metastasis in tissue specimens from patients with non-small lung cancer. Further investigation is needed to indicate if breast cancer metastasis stimulated by GIRK is mediated by MAP kinase, and is correlated with the clinical aggressiveness of ER (−) breast cancer.

A recent review has indicated that potassium channels may be valuable in cancer therapy (Conti, 2004). Others have indicated that a combination therapy of tamoxifen and potassium channel blockers may be important in treatment of breast cancer due to the effects of potassium channels on increasing breast cancer cell proliferation (Abdul et al. 2003). In addition, small conductance Ca^2+^-activated potassium channels may mediate breast cancer cell migration (Potier et al. 2006). Our results and those of others seem to indicate that blockage of GIRK channels may be important in breast cancer, and more work is needed to further elucidate the signaling pathways involved in GIRK signaling in breast cancer, and to indicate if the signaling pathways associated with these GIRK channels are involved in cancer progression and cell migration, as well as in cell proliferation.
